# Socioeconomic inequalities in dental health services in Sao Paulo, Brazil, 2003–2008

**DOI:** 10.1186/s12913-016-1928-y

**Published:** 2016-12-07

**Authors:** Camila Nascimento Monteiro, Mariëlle A. Beenackers, Moisés Goldbaum, Marilisa Berti de Azevedo Barros, Reinaldo José Gianini, Chester Luiz Galvão Cesar, Johan P. Mackenbach

**Affiliations:** 1 Department of Preventive Medicine, Faculty of Medicine, University of São Paulo, São Paulo, Brazil; 2 Department of Public Health, Erasmus University Medical Centre, Rotterdam, The Netherlands; 3 Researcher 1C of National Council for Scientific and Technological Development-CNPq, São Paulo, Brazil; 4 Department of Public Health, School of Medical Sciences, University of Campinas, Campinas, Brazil; 5 Researcher 1D of National Council for Scientific and Technological Development-CNPq, São Paulo, Brazil; 6 Catholic University of São Paulo, Sorocaba, São Paulo Brazil; 7 Department of Epidemiology, Faculty of Public Health, University of São Paulo, São Paulo, Brazil

**Keywords:** Dental health services, Dental care, Inequalities in health care services, Health surveys

## Abstract

**Background:**

Access to, and use of, dental health services in Brazil have improved since 2003. The increase of private health care plans and the implementation of the “Smiling Brazil” Program, the largest public oral health care program in the world, could have influenced this increase in access. However, we do not yet know if inequalities in the use of dental health services persist after the improvement in access. The aims of this study are to analyze socioeconomic differences for dental health service use between 2003 and 2008 in São Paulo and to examine changes in these associations since the implementation of the Smiling Brazil program in 2003.

**Method:**

Data was obtained via two household health surveys (ISA-Capital 2003 and ISA-Capital 2008) which investigated living conditions, lifestyle, health status and use of health care services. Logistic regression was used to analyze associations between socioeconomic factors and dental services use. Additionally, trends from 2003 to 2008 regarding socioeconomic characteristics and dental health service use were explored.

**Results:**

Overall, dental health service use increased between 2003 and 2008 and was at both time points more common among those who had higher income, better education, better housing conditions, private health care plans and were Caucasian. Inequalities in use of dental health care did not decrease over time. Among the reasons for not seeking dental care, not having teeth and financial difficulty were more common in lower socioeconomic groups, while thinking it was unnecessary was more common in higher socioeconomic groups.

**Conclusions:**

The Brazilian oral health policy is still in a period of expansion and seems to have contributed slightly to increased dental health service use, but has not influenced socioeconomic inequalities in the use of these services. Acquiring deeper knowledge about inequalities in dental health service use will contribute to better understanding of potential barriers to reducing them.

**Electronic supplementary material:**

The online version of this article (doi:10.1186/s12913-016-1928-y) contains supplementary material, which is available to authorized users.

## Background

The health care system in Brazil is dynamic and complex. The public sector coverage is universal and aims to provide vaccination, general practice, medical specialists, hospital care, pharmaceutical care, dental care, and other health care services to all [1]. The Brazilian Unified Health System (Sistema Único de Saúde-SUS) aims to provide comprehensive, universal, preventive and curative care through the provision of health services [1]. The system includes services and financial cover from both the public and the private sector in which the private sector offers supplementary coverage of health care services [1]. The difficulty the public sector has in covering the whole population has prompted the development and increase of private health plans. In Brazil, two concomitant phenomena occurred in the 2000s in the context of dental health services: the increase of private dental plans and the appearance of oral health on the political priorities agenda of the federal government [2]. Brazilian national health policies from 2003 to 2008 prioritized key problems in the Brazilian health system [3], and included initiatives such as the Smiling Brazil program (programa Brasil Sorridente).

The Smiling Brazil program, the largest public oral health care program in the world [4], was launched in the period between 2003 and 2006. It aims to improve access to dental care at all levels of complexity, including preventive oral health services, basic oral health services and complex rehabilitation oral health services [3, 5]. Additionally, it aims to increase the quality of care for all dental health problems and decrease inequalities in dental services use [3, 5, 6]. Smiling Brazil promoted an increase in dental health care teams, which usually consists of a dentist, an oral health technician and an oral health assistant [4]. Between 2002 and 2008, the number of dental health teams increased by 407% (from 4261 in 2002 to 17,349 in 2008) and the coverage of dental health teams has increased from 15 to 45% of the population [3]. Nowadays, 24,243 dental health teams operate in 5013 cities in Brazil, covering a total population of over 81 million people [4]. Oral health is a government priority [3, 5] and an integral part of the Brazilian public health system, which was defined by the principles of universality, integrality and equity [7].

Policies to reduce inequalities are necessary in Brazil and particularly in São Paulo, which has considerable, persistent and rising income disparities. The Gini Coefficient in São Paulo was 0.57 in 1991, 0.62 in 2000 and 0.65 in 2010 [8] (the Gini coefficient is a number from 0 to 1 with higher scores indicating more inequality [9]). There are considerable socioeconomic inequalities in dental health service in Brazil [10]. In 2007, only 16.5% of the lower education groups visited dental services, compared with 64.3% of the higher education groups [11]. Previously identified determinants of these inequalities include geographic and social inequalities in the supply of health care services and other determinants of health such as individual lifestyle factors, social and community networks and socioeconomic, cultural and environmental conditions [7, 12, 13].

The use of dental services is influenced by the availability of those services, the geographic distribution of dentists [14], and the resources of the health service to fit the needs of communities [15] and particular groups such as adults and the elderly. Manfredini et al. [2] found that São Paulo offered poor access to public dental care during the period 2000 to 2009, with reduced supply of services to adults and older people. Cost is another important barrier to using dental health services [6, 14] especially for low-income groups [14, 15]. In addition, most private practices are also incompatible with the needs of low-income communities [15]. The Smiling Brazil program provides free dental care and aims to remove this barrier [5, 6]. However, although we know the policy achieved an increase in access to dental care services, it is still unclear who benefited most from this increased access and whether the national oral health policy actually succeeded in decreasing socioeconomic inequalities in the use of these health care services.

The present study aims to analyze socioeconomic differences in the use of dental health services in São Paulo in 2003 and 2008 in adults and the elderly. In addition, the study will explore whether these inequalities changed in the period between 2003 and 2008 during which the Smiling Brazil program was implemented.

## Methods

### Study population

Data have been taken from the population-based health surveys ‘Inquéritos de Saúde no Município de São Paulo’ (ISA-Capital), carried out in 2003 and in 2008, which are based on a representative sample of non-institutionalized residents from São Paulo-Brazil. They comprised two cross-sectional population-based household surveys which investigated living conditions, lifestyle, health status and use of health care services.

Both surveys were based on similar probability samples. The only differences were the number of randomly selected census tracts and the sampling errors. The two-stage sampling was performed within census tracts (primary sampling unit) and households (second stage) [16, 17]. In 2003, 60 sectors were randomly sampled from the 264 census tracts previously selected by the Brazilian Institute of Geography and Statistics (IBGE) [18] for the Brazilian Household Survey. In 2008, 70 sectors were randomly sampled from the 267 census tracts selected by the IBGE [19] for the Brazilian Household Survey. First, the census tracts were stratified according to educational level (lower, middle, higher), defined by the educational level of the head of each household, then the desired number of tracts were selected. In the second stage, households were sampled from the selected tracts in order to obtain an adequate sample size for each domain. The domains were based on gender (male, female) and age group (<1 year, 1–11 years, 12–19 years, 20–59 year, >60 years). A stratified sample of 420 respondents in each domain was approached for interview. The response rate was 78.62% in 2003 and 76.41% in 2008. A total of 3357 respondents were interviewed in 2003 and 3271 in 2008.

Data were collected through a structured questionnaire with mostly closed questions Additional file 1. All interviews were conducted by trained staff who were supervised throughout the study. For quality control, another interview by phone or face to face was carried out in a random sample of 5% of the interviews. The Research Ethics Committee of the University of São Paulo approved the design and conduct of the study. The design and characteristics of ISA-Capital 2003 and ISA-Capital 2008 have been described in detail in: http://www.fsp.usp.br/isa-sp.

### Demographic and socioeconomic factors

The following demographic factors were examined: age (20–59 years and 60 or over) and gender. The present study examines adults and the elderly. There is a reduced supply of services and considerable inequalities in the use of dental services by these groups [2]. We are not studying children or teenagers since they have different oral needs and require specific oral policies with more focus on the prevention of oral diseases [4].

The socioeconomic factors studied were: ethnicity (Caucasian, when respondent reported white, and non-Caucasian, when the respondent reported black/mixed/other), education (0–3 years of study, 4–7, 8–11 and 12 or more), household income (≤1 minimum legal wage (mlw), >1 to 2.5 mlw, ≥ 2.5 to 6 mlw, ≥6 mlw), housing condition and having a private health plan. Assuming that ethnicity might be a basis for social differentiation in Brazil [20] we included ethnicity as a socioeconomic indicator. The minimum legal wage (monthly) in 2003 was R$240.00 ($68.00^a^) and in 2008 R$415.00 ($233.00^b^). Monthly family income per head was not adjusted for inflation but was used to rank each individual at each year along the income distribution and is treated as a relative measure of social position in each time period. Housing condition was divided into adequate or inadequate; it was considered adequate when the house had piped water from the public network, had electric lighting, was connected to the sewer system and had an internal lavatory, and when the street had street lighting. If any of these factors were missing, the housing was considered inadequate.

- ^a^Dollar value on 01/01/2003: R$3.53
- ^b^Dollar value on 01/01/2008: R$1.78

### Outcome measures

- *Use of dental health services*: The study measured dental services use and focused on variations in use across socioeconomic groups. All respondents were asked whether they had been seen by a dentist in the last 12 months. Those who responded positively were categorized as “used dental services in the last 12 months”. The others were categorized as “did not use dental services in the last 12 months”. Those respondents who reported not having seen a dentist in the past 12 months were asked why not. Reasons for not seeing a dentist were itemized in a list of options and included: ‘financial difficulty’, ‘I have no teeth’, ‘I did not think it was necessary’, ‘I did not have time’, ‘the dental care service was full’, ‘the dental care service is too far from my home’, and ‘I did not know the service was available’.
- *Use of dental health services in the public sector*: To identify whether dental care services were financed by the public sector or the private sector (e.g. private health plan), respondents who used dental services were asked which sector covered the services they visited.

### Statistical analyzes

The samples ISA-Capital 2003 and ISA-Capital 2008 were weighted to compensate for the different selection probabilities. The weighting was performed at an individual level, the sectors were weighted for the use of different sampling fractions in the strata. The trends from 2003 to 2008 regarding socioeconomic characteristics and use were analyzed by Chi-square tests. Crude and adjusted (by age and gender) logistic regressions were used to explore the associations between socioeconomic factors and the different measures related to use. The dental health service use difference between 2003 and 2008 was analyzed by interactions of time and each socioeconomic factor. The interactions in the logistic regression model were tested for their departure from multiplicativity. The reasons for not seeking a dentist in relation to educational level and income were analyzed by Chi-square tests. Significance was judged with α = 0.05. Analyses were carried out in Stata 12.0 using the survey package, which considers the effects of complex study design and allows the different weights of the observations to be embedded.

## Results

Table 1 shows the descriptive results of the sample and trends from 2003 to 2008. The sample in 2008 was slightly better educated and included more non-Caucasian people. The samples were comparable with respect to age and gender composition.

**Table 1:** Characteristics of the ISA-Capital 2003 and ISA-Capital 2008 respondents living in the city of São Paulo, Brazil

	2003	2008	Trend 2003–2008
Total sample	1667	2086	
Sociodemographic factors			
Age group	%^a^ (n)^b^	%^a^ (n)^b^	
20–59	84.0 (795)	83.7 (1162)	
60+	16.0 (872)	16.3 (924)	
Gender			
Male	45.1 (803)	46.3 (848)	
Female	54.9 (864)	53.7 (1238)	
Socioeconomic factors			
Ethnicity			<0.001^c^
Caucasian	67.42 (1077)	62.2 (1311)	
Non-caucasian	32.6 (542)	37.8 (770)	
Education (years of study)			0.001^c^
0–3	14.2 (450)	10.2 (413)	
4–7	25.7 (509)	19.0 (591)	
8–11	36.2 (450)	46.6 (774)	
12+	23.9 (231)	24.2 (305)	
Household income (minimum legal wage)			0.356^c^
* ≤1*	12.5 (262)	9.9 (241)	
* >1 to 2.5*	27.6 (386)	38.6 (628)	
* ≥2.5 to 6*	32.3 (434)	31.4 (459)	
* ≥6*	27.6 (251)	20.1 (211)	
Housing condition			0.199^c^
Adequate	83.1 (1410)	88.2 (1805)	
Inadequate	16.9 (257)	11.8 (281)	
Dental health service use			
Dental health service use	46.7 (611)	55.4 (1002)	0.001^c^
Dental health service use in public sector	5.8 (54)	6.8 (91)	0.516^c^

^a^Percentages weighted according to the weight given in the sampling process

^b^Unweighted frequency

^c^Difference between proportions in 2003 and proportions in 2008 Chi-square Pearson

There was an increase in the proportion of dental health service use from 2003 to 2008 (Table 1). The increase in use was present across all socioeconomic groups including the lowest socioeconomic groups (Table 2). The use that was covered by the public sector did slightly increase, but the increase was not significant (Table 1). The increases in use from the public sector were also not clearly located within a certain socioeconomic group (Table 3).

**Table 2:** Crude and adjusted logistic regression for dental service use and socioeconomic factors. São Paulo, 2003 and 2008

	Dental health service use in the last 12 months 2003			Dental health service use in the last 12 months 2008			Difference in association of socioeconomic factor and dental service use between 2003 and 2008^a^		Dental health service use difference between 2003 and 2008^b^
	Dental service use % (*n*)	Crude OR (95% CI)	Adjusted OR^c^ (95% CI)	Dental service use % (*n*)	Crude OR (95% CI)	Adjusted OR^c^ (95% CI)	Crude Model- *p*-value	Adjusted Model- *p*-value	*p*-value
Education							0.844	0.480	
0–3	23.3 (86)	1	1	29.3 (112)	1	1			0.005
4–7	35.1 (159)	1.8 (1.1–3.0)	1.7 (1.1–2.9)	38.2 (219)	1.5 (1.1–2.0)	1.5 (1.1–2.0)			0.002
8–11	48.7 (206)	3.1 (1.8–5.3)	2.9 (1.7–5.1)	58.8 (445)	3.4 (2.5–4.8)	3.4 (2.4–4.8)			<0.001
12+	71.0 (155)	8.1 (4.3–15.0)	7.6 (3.9–5.1)	73.2 (224)	6.6 (4.4–10.0)	6.6 (4.3–10.1)			0.009
Household income							0.147	0.102	
* ≤1*	32.8 (58)	1	1	39.3 (71)	1	1			0.004
* >1 to 2.5*	34.7 (84)	1.1 (0.7–1.8)	1.0 (0.6–1.7)	47.5 (263)	1.4 (0.9–2.2)	1.3 (0.8–2.0)			<0.001
* ≥2.5 to 6*	42.2 (211)	1.5 (0.9–2.3)	1.4 (0.9–2.2)	59.4 (253)	2.3 (1.3–3.8)	2.1 (1.3–3.5)			<0.001
* ≥6*	58.9 (258)	2.9 (1.7–5.1)	2.8 (1.6–4.8)	69.3 (141)	3.5 (2.1–5.9)	3.3 (2.0–5.4)			0.023
Housing condition							0.655	0.443	
Adequate	49.9 (544)	1	1	57.2 (902)	1	1			0.014
Inadequate	30.9 (67)	0.4 (0.3–0.8)	0.4 (0.2–0.7)	41.5 (100)	0.5 (0.4–0.7)	0.5 (0.4–0.7)			<0.001
Ethnicity							0.982	0.631	
Caucasian	51.3 (440)	1	1	60.6 (683)	1	1			<0.001
Non-Caucasian	37.2 (166)	0.6 (0.4–0.8)	0.5 (0.4–0.7)	46.6 (316)	0.6 (0.4–0.7)	0.5 (0.4–0.7)			<0.001
Private Health Plan									
Private health plan-No				45.4 (445)	1	1			
Private health plan-Yes				65.7 (557)	2.3 (1.8–2.9)	2.4 (1.9–3.1)			

^a^Results of the interaction analyses between time and socioeconomic factor. For the models including education and income, likelihood ratio tests were used to obtain an overall significance test for the interaction term

^b^Difference between proportions in 2003 and proportions in 2008. Chi-square Pearson

^c^Adjusted for age and gender

**Table 3:** Crude and adjusted logistic regression for use of dental service in the public sector. São Paulo, 2003 and 2008

	Dental health service use in the public sector-2003			Dental health service use in the public sector-2008			Difference in association of socioeconomic factor and dental service use between 2003 and 2008^a^		Dental health service use from public sector- 2003 and 2008^b^
	Dental service use from public sector % (*n*)	Crude OR (95% CI)	Adjusted OR^c^ (95% CI)	Dental service use from public sector % (*n*)	Crude OR (95% CI)	Adjusted OR^c^ (95% CI)	Crude Model- *p-value*	Adjusted Model- *p-value*	*p*-value
Education							0.098	0.075	
0–3	19.0 (21)	1	1	14.9 (20)	1	1			0.048
4–7	9.9 (18)	0.5 (0.2–0.9)	0.5 (0.2–0.9)	15.00 (31)	1.0 (0.5–2.1)	1.1 (0.5–2.2)			0.865
8–11	6.2 (13)	0.3 (0.1–0.8)	0.3 (0.1–0.9)	6.9 (36)	0.4 (0.2–0.8)	0.4 (0.2–0.8)			0.201
12+	0.5 (2)	0.02 (0.01–0.1)	0.02 (0.01–0.2)	2.0 (4)	0.1 (0.04–0.4)	0.1 (0.04–0.8)			0.248
Household income^d^							0.084	0.069	
* ≤1*	10.5 (9)	1	1	14.5 (21)	1	1			0.255
* >1 to 2.5*	16.2 (15)	1.7 (0.5–5.2)	1.9 (0.6–5.9)	8.3 (26)	0.5 (0.2–1.2)	0.5 (0.2–1.3)			0.121
* ≥2.5 to 6*	8.0 (25)	0.7 (0.2–2.9)	0.8 (0.2–3.2)	2.6 (8)	0.2 (0.1–0.6)	0.2 (0.1–0.6)			0.002
* ≥6*	1.4 (5)	0.1 (0.02–0.6)	0.1 (0.03–0.7)	2.4 (7)	0.1 (0.02–0.4)	0.1 (0.02–0.4)			0.804
Housing condition							0.142	0.067	
Adequate	5.4 (45)	1	1	6.3 (79)	1	1			0.668
Inadequate	8.8 (9)	1.7 (0.5–5.8)	1.9 (0.5–6.5)	11.6 (12)	1.9 (0.9–4.2)	1.9 (0.9–4.3)			0.157
Ethnicity							0.800	0.426	
Caucasian	5.2 (32)	1	1	4.7 (44)	1	1			0.995
Non-Caucasian	7.5 (22)	1.5 (0.6–3.3)	1.7 (0.8–3.7)	11.4 (47)	2.6 (1.4–4.8)	2.9 (1.6–5.2)			0.701

^a^Differences in socioeconomic characteristics between 2003 and 2008 among population who used dental services

^b^Difference between proportions in 2003 and proportions in 2008. Chi-square Pearson

^c^Adjusted for age and gender

^d^Missings were excluded

Table 2 shows that dental health service use, adjusted for age and gender, was associated with higher education, higher income, adequate housing conditions and being Caucasian in 2003 and in 2008. In 2008, use of dental health services was also associated with having a private health plan (not measured in 2003). The proportion of people who used dental health services increased between 2003 and 2008 for all socioeconomic groups, including the lower socioeconomic groups. However, inequalities in dental health services remained similar in this period. No significant interactions were observed for time for any socioeconomic factor, in the crude and adjusted models.

The way in which dental services were paid for, was also socioeconomically patterned; the lower socioeconomic groups were much more often covered by the public sector than the higher socioeconomic groups (Table 3).

The reasons for not attending dental services in 2003 and 2008 are shown in Table 4, according to education and income. The reasons ‘not having teeth’ and ‘financial difficulties’ were reported more often by lower-educated and lower- income respondents. There has been a strong decline in financial difficulty as the main reason for not using dental care in the lower socioeconomic groups between 2003 and 2008. The reason ‘did not think dental care was necessary’ was more often reported in higher-income respondents in 2003 and 2008. In this same period, there was an increase in the proportion of respondents with lower education and lower income who did not visit a dentist because they did not think it was necessary.

**Table 4:** Main reasons for people not seeking dental services in the previous 12 months and socioeconomic characteristics of education and income. São Paulo, 2003 and 2008

Reason	2003			2008		
	0–7 years of study % (*n*)	7 years or more % (*n*)	Difference between educational groups for each reason: *p*-value	0–7 years of study % (*n*)	7 years or more % (*n*)	Difference between educational groups for each reason: *p*-value
Financial difficulty	42.6 (190)	25.0 (64)	<0.001	33.3 (165)	29.3 (104)	0.005
‘I have no teeth’	17.0 (216)	10.9 (71)	<0.001	19.4 (216)	9.4 (76)	<0.001
‘I did not think necessary’	24.8 (193)	45.7 (130)	<0.001	33.5 (210)	40.8 (154)	0.066
‘I did not have time’	7.4 (26)	6.6 (16)	0.125	6.5 (26)	13.0 (38)	0.450
‘The dental care service was full’	2.6 (11)	1.0 (2)	0.231	1.8 (8)	2.2 (9)	0.689
‘The dental care service is too far from my home’	0.05 (2)	0	n. a^b^	0.6 (4)	0.1 (1)	n. a^b^
‘I did not know the service was available’	0.03 (1)	0	n. a^b^	0.3 (2)	0.2 (1)	n. a^b^
	*<*2.5 mw % (*n*)	*≥*2.5 mw % (*n*)	Difference between income groups for each reason: *p*-value	<2.5 mw % (*n*)	≥2.5 mw % (*n*)	Difference between income groups for each reason: *p*-value
Financial difficulty	44.3 (142)	23.2 (68)	<0.001	38.0 (168)	20.0 (53)	<0.001
‘I have no teeth’	15.8 (144)	10.1 (84)	<0.001	15.0 (157)	10.9 (78)	0.0005
‘I did not think necessary’	25.6 (134)	46.3 (158)	<0.001	31.9 (185)	47.4 (134)	0.0007
‘I did not have time’	5.3 (15)	9.1 (22)	0.552	8.5 (31)	14.5 (30)	0.713
‘The dental care service was full’	2.6 (9)	0.8 (2)	0.150	2.0 (9)	2.3 (6)	0.751
‘The dental care service is too far from my home’	0.2 (1)	0	n. a^b^	0.4 (4)	0	n. a^b^
‘I did not know the service was available’	0.03 (1)	0	n. a^b^	0.5 (3)	0	n. a^b^

^a^Differences in reason for not seeking dental services between 2003 and 2008

^b^Numbers too small to analyze

## Discussion

In both 2003 and 2008 dental health service use in adults and older people was greater among those with higher education, higher income, adequate housing condition and Caucasians. In 2008, the dental health service use was greater among those with a private health plan. Two important reasons for not seeking dental care which were more common in lower socioeconomic groups were not having teeth and financial difficulty. Higher socioeconomic groups reported ‘I did not think it was necessary’ as a reason for not visiting the dentist. Dental health service use increased between 2003 and 2008 in all socioeconomic groups, but inequalities in the use of these services did not decrease over time.

### Strengths and limitations of the study

The deeper knowledge about inequalities in use provided by this study is important to facilitate the formulation of appropriate public policies and to provide better understanding of the potential barriers to reducing inequalities in health care services use. However, some limitations should be borne in mind when interpreting the results of this study. Due to small number of people who reported dental service use from public sector (5.80% in 2003 and 6.82% in 2008), some results were relatively imprecise. Since the questionnaire does not have questions about dental care needs, we were not able to adjust for different needs across socioeconomic groups. However, by adjusting for age and gender, we did try to minimize bias due to need across all socioeconomic groups. Although there are different private health plans, we were not able to analyze the quality of these plans; thus we were not able to discuss the quality of dental health care provided by the private sector and compare it with that provided by the public sector in this study. This would be very interesting since there may be large quality differences between services delivered to different socioeconomic groups. Finally, ISA-Capital 2008 does not specifically inquire whether the use of dental health services was due to the newly implemented national oral health policy and we can therefore not know for sure whether the observed increase of dental health service use is specifically due to this policy. There could be other changes in society that have biased the results. However, since the Smiling Brazil program was very important in Brazil to increase access to dental health services and there were large changes related to dental care services within the timeframe of the study, we are confident that this program at least partly contributed to the observed changes.

### Use of dental health service, socioeconomic inequalities and trends 2003–2008

Previous studies have shown that the use of dental health services is more common in more affluent socioeconomic groups [14, 21, 22]. This was confirmed in our study since we found that the use of dental health services was greater in those who had higher income, higher education, adequate housing conditions and who were Caucasian.

Additionally, our study found that, in 2008, the use of dental health services was greater among those with a private health care plan than those in the population who do not have a private health care plan and thus rely on public sector coverage. Almost half the population in São Paulo had a private health plan in 2008 [9]. In general, people with a private health plan have higher education, higher income, more often have adequate housing condition and are more often Caucasian [9]. These socioeconomic disparities in the distribution of private health plans can also been seen in the different regions of São Paulo; in the more affluent regions, a large majority of the population is covered by private health plans while in the more deprived areas, almost everyone relies on public health care coverage [9, 23]. Nowadays, in more deprived areas in São Paulo, there are private dental health plans specifically for the low-income population, which is increasing access to dental health care by this population [2, 9, 23].

The lower socioeconomic groups were much more often covered by the public sector than the higher socioeconomic groups and the use of by public sector scarcely increased from 5.8% in 2003 to 6.8% in 2008. This confirms the need for expansion of the existing national oral health policy, as reported in the study by Manfredini et al. [2]. According to Wallace and MacEntee [15], there is a poor fit between private practice dentistry, public dental benefits and the oral health needs of low-income communities. In countries with public coverage on dental health services, there are less socioeconomic inequalities in use of dental health services, comparing to countries without public coverage [24]. However, despite the increase in access and public coverage in Brazil, inequalities in the use of dental care still persist in Sao Paulo, which could indicate that further improvements in the care and coverage system are necessary.

The inequalities in use of dental care services may also reflect socioeconomic inequalities in dental health in Brazil and particularly in São Paulo. Our study shows that the reasons ‘not having teeth’ and ‘financial difficulties’ were reported more often by lower educated and lower income respondents. The reason ‘did not think the dental care was necessary’ was more often reported in higher income respondents in 2003 and in 2008. The high percentage of toothless people in this population of adults and the elderly, especially in the lower socioeconomic groups, may indicate historical inequalities in dental care use; these people may have needed to use dental health service in the past but did not have access at that time and this may have contributed to the loss of teeth. In the past, tooth loss was often believed to be a natural phenomenon [25]. However, today it is seen as a something that can and should be prevented by health policies [25]. Some studies have explored the trends in social inequality in tooth loss [26, 27] and found high percentages of toothless people in lower socioeconomic groups, which confirms the results of the present study.

There was an increase of use of dental health services between 2003 and 2008 in São Paulo: from 46.8% in 2003 to 55.4% in 2008. The increase followed a trend: in 1990, use of dental health services in the previous 12 months in the São Paulo metropolitan area was 31.8% [28]. The increase of dental service use between 2003 and 2008 was significant across all socioeconomic groups, including the lowest socioeconomic groups. The increasing of income of the population and the increase in private dental health plans may have contributed to the increase in dental service use. The implementation of the Smiling Brazil program may have contributed to the slight increase in dental service use from public sector in the lowest income group, the non-Caucasians and the population with inadequate housing condition between 2003 and 2008. However, these increases were not significant and we did not see an increase in use from public sector in the lowest educational groups. Compared to other policies, such as the immunization program, the Smiling Brazil program is new and in a period of expansion. Its implementation indicates numerical expansion and extended health care coverage [5] and improved the organization and planning of oral health services [4]. The dental care use has increased in all socioeconomic groups, including the lower socioeconomic groups but there were persistent socioeconomic inequalities in the use of dental health services in 2003 and 2008, despite the aim of the oral health policy to decrease inequalities [5] and the favorable indications regarding the effectiveness of this policy in reducing disparities in oral health [29]. The lack of significant improvements in decreasing inequalities may be partly due to the small window between the implementation (2003–2006) and our time of measurement (2008). However, since 2008, according the Brazilian National Health Survey [30], the frame did not change: only 53.9% used dental services in the previous 12 months in São Paulo state in 2013 and was higher in higher education groups (73.4%) compared to lower education groups (46.3%). Also, Scherer and Scherer [31] analyzed oral health work changes in primary health care after a decade of the “Smiling Brazil” Program and found very few changes in oral health work. The population-based health survey ISA-Capital 2015^1^ is almost completed and will provide monitoring on access and use of dental health services in São Paulo city.

Potential problems of the policy may include a lack of regular supply of materials to health facilities and maintenance of dental equipment, in addition to the inability to pay the full contract personnel in health facilities [32]. Structural problems in the Brazilian health system such as the limited funding [3, 32], the disjunction between the public and private sectors [32] and the fragmentation of policies represent challenges to the implementation of universal access to dental health services from the public sector and the decrease of inequalities in dental health service use.

## Conclusions

Brazil is one of the few large countries with universal health care coverage. However, one of the challenges faced by the Brazilian Health System is the need to decrease socioeconomic health disparities between individuals. The Smiling Brazil program is in a period of expansion and, although the increase in use of dental services from public sector was not significant, the program may have contributed to the overall increase in dental health service use in the period 2003–2008 in São Paulo. However, until 2008 the policy has not yet achieved a decrease in the existing socioeconomic inequalities in dental health service use.

## Additional file

Additional file 1: Questionnaire ISA-Capital 2003 and ISA-Capital 2008. Data of this study were collected through a structured questionnaires with mostly closed questions named Questionnaire ISA-Capital 2003 and ISA-Capital 2008. (DOC 73 kb)

## Abbreviations

ESF: Brazil’s Family Health Programme
IBGE: Brazilian Institute of Geography and Statistics
ISA-Capital: Population-based health surveys ‘Inquéritos de Saúde no Município de São Paulo’
SUS: Brazilian Unified Health System

## Footnotes

1 ISA-Capital 2015: https://www.fsp.usp.br/isa-sp.
