# Supplementary material for: Socioeconomic inequalities in dental health services in Sao Paulo, Brazil, 2003–2008
Source: BMC Health Serv Res. 2016 Dec 7;16:683. doi: 10.1186/s12913-016-1928-y (PMC5142432; doi:10.1186/s12913-016-1928-y)
Supplement: Additional file 1: — Questionnaire ISA-Capital 2003 and ISA-Capital 2008. Data of this study were collected through a structured questionnaires with mostly closed questions named Questionnaire ISA-Capital 2003 and ISA-Capital 2008. (DOC 73 kb) [file 12913_2016_1928_MOESM1_ESM.doc]

SUPPLEMENTARY FILE: QUESTIONNAIRE ISA-CAPITAL

**ISA-CAPITAL 2003 AND ISA-CAPITAL 2008**

**QUESTIONNAIRE**

**ACRONYMS:**

SUS: Brazilian Unified Health System

ESF: Brazil`s Family Health Programme

BLOCK A-LIST OF RESIDENTS

BLOCK B - CONTROL

BLOCK C - MORBIDITY (MORBIDITY IN TWO WEEKS, CHRONIC DISEASES, DISABILITIES)

BLOCK D - ACCIDENTS AND VIOLENCE (TRAFFIC ACCIDENT, FALLS, VIOLENCE)

BLOCK E - EMOTIONAL HEALTH

BLOCK F - QUALITY OF LIFE

BLOCK G - USE OF HEALTH SERVICES

BLOCK G1 – VISITING DOCTOR

BLOCK G2 - HOSPITALIZATIONS

BLOCK G3 - HEALTH PLAN

BLOCK G4 – UTILIZATION OF SUS / ESF

BLOCK G5 - DENTIST

BLOCK H - PREVENTIVE EXAMS

BLOCK I - IMMUNIZATION (HEPATITIS V, FLU, PNEUMONIA, TETANUS)

BLOCK J - MEDICATION

BLOCK K – FOOD, PHYSICAL ACTIVITY, SMOKING, ALCOHOLIC BEVERAGES

BLOCK L/M/N - SOCIO-ECONOMIC AND SOCIO-DEMOGRAPHIC CHARACTERISTICS

BLOCK O - HEALTH EXPENSES

BLOCK P - PRESENCE OF ANIMALS

**HEALTH SERVICES UTILIZATION**

G1 01. When was the last time that you sought a Health Service?
1. Less than 2 weeks
2. Between 15 days and one month
3. More than 1 month to 3 months
4. Longer than 3 months to 6 months
5. Longer than 6 months to 1 year
6. Over a year
9. Not answer or Don`t know

G1 02. How many times you was in the Health Service in the last 30 days?
| __ | __ | Times

REGARDING THE LAST TIME THAT LOOKED FOR SOME HEALTH SERVICE:

G1 10 The Health Service is:
1. Public 2. Private

9. Not answer or Don`t know

G1 11.You gotta be serviced?
1. No
2. Did not, but scheduling was done
3 yes.
9. Not answer or Don`t know

G1 12. You could be seen at the service sought?
1. Was directly seen without first schedule
2. Previously scheduled consultation
3. Was assisted by the Family Health Program
4. Was referred by another service or professional
9. Not answer or Don`t know

G1 18. Who covered the Health Service spending?
1. SUS
2. Health Plan
3. Interviewee
4. Other:________________
9. Not answer or Don`t know

**C24 What do you think about the Health Service?**

Very good……………..1

Good…………………….2

Regular………………..3

Bad……………………..4

Not answer or Don`t know……9

**C25 Why not seek the Heath Service?**

Financial problem………………………………..1

Difficult geographic Access…………….……2

Think not necessary……………………..…….3

Don`t have time to go to HS…………….….4

Don`t know where can go…………………..5

The problem was solved with ESF……..6

Other…………………………………………..…….7

Not answer or Don`t know………….…9

**C26 Why do you not been serviced in health Service? (c2611)**

Don`t have vacancy………………………………….…….1

Doens`t have doctor in HS………………………………2

Doesn`t have other Health professional…………..3

The machine is not working…………………….………4

Financial problem………………………………………..…..5

Waiting for a long time and back down…………..6

Other………………………………………………………..…….7

Not answer or Don`t know………………………...…9

**C30 The problem was solved?**

No……………………1

Yes…………………2

Yes, partly………3

Not answer or Don`t know………………………...…9

**DENTIST**

G5 08. When you consulted a dentist last?
1. Less than 6 months
2. Between 6 months and 1 year
3. Between one year and 2 years
4. Between 2 years and 3 years
5. 3 years or more
6. Never
9. Not answer or Don`t know

G5 09. Why you did not consult a dentist in the last 12 months?
1. Financial Difficulty
2. Difficulty of being assisted in Health Service
3. Did not think necessary
4. Had no time
5. Afraid
6. Uses denture
7. Has no teeth

9. Not answer or Don`t know

G5 13. You could be seen at the service sought?
1. Was directly to Health Service without first schedule
2. Previously scheduled
3. Was assisted by PSF/ESF
4. Was referred by another Health professional
9. Not answer or Don`t know


G5 17. Who covered the expenses?
1. SUS
2. Health Plan
3. Interviewee
4. Other:________________
9. Not answer or Don`t know

**HEALTH PLAN**

G3 01. You have or had Health Plan (medical or dental)?
1. Not
2. Yes
3. Yes, I had*****
9. Not answer or Don`t know

* G3 02. How long you has failed to have the Health Plan?
______ Years ______ months

9. Not answer or Don`t know

G3 08. You already needed some Health Care and your Health Plan did not cover?
1. No
2. Yes What ______________________________________________
9. Not answer or Don`t know

**HEALTH SPENDING**

How the family spent on Health in the last month?

| Medicines | 1.No expenses | 2.R$____________ | 99999. Don`t answer or Don`t know |
| --- | --- | --- | --- |
| Doctor visiting | 1.No expenses | 2.R$____________ | 99999. Don`t answer or Don`t know |
| Other professional of health visiting | 1.No expenses | 2.R$____________ | 99999. Don`t answer or Don`t know |
| Hospitalization | 1.No expenses | 2.R$____________ | 99999. Don`t answer or Don`t know |
| Home care | 1.No expenses | 2.R$____________ | 99999. Don`t answer or Don`t know |
| Other:________ | 1.No expenses | 2.R$____________ | 99999. Don`t answer or Don`t know |

**UTILIZATION OF SUS AND ESF**

G4 01.You used any Health Service in SUS (visiting doctor, vaccination, take medicines, etc..)

1. not 2. Yes 9. 9. Not answer or Don`t know

G4 02. Which was the last Health Service that you used in SUS?
1. Visiting Doctor
2. Hospitalization
3. Vaccination
4. To take medicines
5. Laboratory exams
6. Ambulatory (measuring pressure, blood glucose, etc.)
7. Other
9. Not answer or Don`t know

G4 06. You know the Family Health Program (PSF or ESF)?
1. No 2. Yes 9. Not answer or Don`t know

G4 05.You or someone in your family received visits from workers from PSF/ESF ?
1. No. 2. yes 9. Not answer or Don`t know

G4 07. You already used the services offered by the PSF/ESF?
1. No. 2. yes 9. Not answer or Don`t know

SOCIOECONOMIC AND SOCIODEMOGRAPHIC PROFILE

| B 06. Date of Birth: |__|__| / |__|__| / |__|__|__|__| | B 07. Gender 1. male |  | 2. female |
| --- | --- | --- | --- |

L 01. Which is your race?
1. White
2. Black
3. *Japanese (yellow)*
4. Mulatto
5. Indigenous
6. Other:___________________________
9. Not answer or Don`t know

L 03. Which is your religion?
01. None
02. Evangelical or Protestant
03. Catholic
04. Spiritualist
05. Judaism
06. Buddhism
07. Umbanda / Candomblé
08. Islamism
09. Other
99. Not answer or Don`t know

L 06. Where You born?
1. São Paulo
2. In another city in the state of São Paulo
3. In another state: _____________________________
4. 4 in another country
9. Not answer or Don`t know

L10 What your marital status?
1. Married *(in civil)*
2. Lives together
3. Separated
4. Widower
5. Single
9. Not answer or Don`t know
L 16. Education (years of study)
01. Cannot read and write

02. 4-7 years of study

03. 8-12 years of study

04. 13 or more years

9. Don`t answer or don`t know.

L 19. Currently you exercises any gainful work activity?
1. not 2. Yes 9. Not answer or Don`t know

If the interwiee asked 2 in L19: L 20 What is your current situation regarding work and activities:.

1. Retired
2. Pensioner
3. Housewife
4. Student
5. Unemployed
9. Not answer or Don`t know


L 23. Which is (or was) your occupation in your main job?
_______________________________

L 24. In his main work, the (a) Mr. (a) is (was) employee, employer or own account?
01. Employee of the private sector
02. Employee of the public sector (including mixed capital companies)
03. Their own or with unattended property
04. Account or own property without autonomic
05. Employer with up to 4 employees
06. Employer with 5 or more employees
07. Domestic worker
9. Not answer or Don`t know

L 28. Which was your net income last month with the (main) job?
R$ | __ | __ | __ | __ | __ |, 00 99999. Not answer or Don`t know

L 29. You work in another job?

1. not 2. Yes 9. Not answer or Don`t know

L 33. Considering all their income (occupations, pensions, and other), what was your overall net income last month?
R$ | __ | __ | __ | __ | __ |, 00 99999. Not answer or Don`t know

G 34 Doing a *rough* calculation, the average of income last month goes:
1. Less than 1 minimum salary (ms)
2. Between 1 and 2 ms
3. Between 3 and 4 ms
4. Between 5 and 9 ms
5. Between 10 and 20 ms
6. Above 20 sm
9. Not answer or Don`t know.

M03. Characterization of home:
1. Brick house, with flooring, roofing tiles or slab with floor, finished
2. Apartment
3. Poor house without a trailer, still not finished, or with dirt floors
4. Shack
5. Rooming house
6. Others
9. Not answer or Don`t know

**Questions M04 to M36: Answer: 1. not 2. Yes 9. Not answer or Don`t know**

M 04. The street where the home is located is paved?

M 05. The street where the home is located has sidewalks and gutters?

M 06. The street where the home is located has street lighting?

M 07. At the household has piped water from the public network at home?

M 08. At the household has electric lighting?

M 09. Connected to the sewer system?

M 10. Has Public garbage collection?

M 11. Has toilet / bathroom inside?

M23. DVD / BluRay

M 24. Dishwasher

M 25. Air conditioner

M 26. Vacuum Cleaner

M 27. Telephone line

M 28. Cellphone

M 29. Camera / Digital Camera

M 30. Computer

M 31. Laptop / tablet

M 32. Motorcycle

M 33. Cycling

M 34. Car

M 35. Have internet access at home?

M 36. This home has the housemaid 3 times a week or more?
